# Predictors of regular mammography use among American Indian women in Oklahoma: a cross-sectional study

**DOI:** 10.1186/1472-6874-14-101

**Published:** 2014-08-28

**Authors:** Eleni L Tolma, Julie A Stoner, Ji Li, Yoonsang Kim, Kimberly K Engelman

**Affiliations:** 1 Department of Health Promotion Sciences, College of Public Health, University of Oklahoma Health Sciences Center, CHB Rm. 473, P.O. Box 26901, 73126 Oklahoma City, OK, USA; 2 Department of Biostatistics and Epidemiology, College of Public Health, University of Oklahoma Health Sciences Center, Oklahoma City, OK, USA; 3 Institute for Health Research and Policy, University of Illinois at Chicago, Chicago, IL, USA; 4 Department of Preventive Medicine and Public Health, University of Kansas School of Medicine, Kansas City, KS, USA

**Keywords:** Breast cancer, Native American, American Indian, Theory of planned behavior, Mammography, Quantitative research

## Abstract

**Background:**

There are significant disparities in breast cancer screening and survivorship between American Indian (AI) and non-Hispanic white women. This study aimed to identify the salient beliefs AI women from Oklahoma have on regular mammography screening, and to determine which beliefs and health- related practices are associated with past mammography screening behavior.

**Methods:**

This study used an integrated model of the Theory of Planned Behavior as the guiding theoretical framework. Data were collected from 255 (mean age = 51 years, SD 7.64 years) AI women randomly selected from a rural Oklahoma medical clinic (response rate: 79%). Multivariate logistic regression was used to identify factors associated with self-reported past mammography within the last two years while controlling for demographic variables. Associations were summarized using odds ratios (OR), the ratio of the odds of past mammography per a 1-unit increase in continuous independent factor scales (subjective physician norm, cultural affiliation, fatalism, knowledge of mammography screening guidelines, and perceived behavior control barriers) or between groups defined by categorical variables, and 95% confidence intervals (CI).

**Results:**

Of the participants, 65% (n = 167) reported a screening mammogram within the last two years. After adjustment for age and educational status, women with a higher total subjective-norm physician score (OR = 1.15, 95% CI: 1.06-1.24), a higher knowledge of mammography screening guidelines (OR = 1.52, 95% CI: 1.00-2.31), a family history of breast cancer (OR = 9.97, 95% CI: 3.05-32.62), or reporting an annual versus none or a single physician breast examination (OR = 5.57, 95% CI: 1.79-17.37) had a higher odds of past mammography. On the other hand, women who were more culturally affiliated (OR = 0.42, 95% CI: 0.24-0.74), perceived more barriers (OR = 0.86, 0.78-0.94), or had higher fatalistic attitudes toward breast cancer (OR = 0.90, 95% CI: 0.82-0.99) had lower odds of past mammography.

**Conclusion:**

In the development of culturally-appropriate interventions promoting mammography among AI communities, emphasis could be put on the following: a) promoting clinic-related practices (e.g. physician recommendation, physician breast examination); b) promoting community-related practices (e.g. knowledge about mammography while eliminating fatalistic attitudes); and c) reducing environmental barriers.

## Background

Breast cancer continues to be the most commonly diagnosed cancer and the second leading cause of death among women in the United States [1]. While breast cancer rates vary by race/ethnicity, socioeconomic status (SES), and geographic region, among American Indian (AI) women breast cancer remains a major cause of death [1]. Although the incidence rates among AI women today are the fourth lowest at 89.1/100,000 from 2005–2009 when compared to non-Hispanic white women at 123.3/100,000 and African American women at 118.0/100,000, mortality rates have declined among all racial/ethnic groups except for American Indians for the most recent 10-year time period [2]. Today, many AI women do not receive breast cancer screening despite its availability. In 2008, only 59.7% of AI women age 40 and over had a mammogram within the past 2 years while the screening rate for non-Hispanic white women was 68% [1].

In Oklahoma, the incidence rate of breast cancer among AI women was 140.5/100,000 compared to 121.5/100,000 among non-Hispanic white women for 2005–2009 [3]. Similarly, disparities still exist regarding late-stage breast cancer diagnosis where 34.2% of the late-stage cases were diagnosed among AI women compared to 31% among Non-Hispanic white women in contrast to the Oklahoma demographic distribution in which AI women comprised 11% of the adult female population aged 18 and older compared to 74% non- Hispanic white based on 2010–2012 census data [3,4]. Therefore, with no doubt, breast cancer is an important public health issue among AI women, not only in the US, but also in Oklahoma.

Numerous barriers exist that prevent AI women from seeking mammography screenings. These limitations include lack of education and awareness of mammography screening [5], reduced access to medical services [6], absence of family history [7], historical trauma [8], embarrassment [9,10], fear [9,11], and oftentimes fatalism [9]. Physician recommendation has been established as women’s primary motivating factor to get a screening mammogram [12,13]. However, only two studies [14,15] showed that physician referral was positively associated with recent mammography experience within the AI population. Other social factors include encouragement by significant others, such as family members, friends, and elderly [16]. Moreover, the impact of traditional Native identity on mammography screening has not been clear. Some studies show that women who are more traditional are more likely to get a screening mammogram [17,18] whereas others do not [19,20].

**Figure 1:**
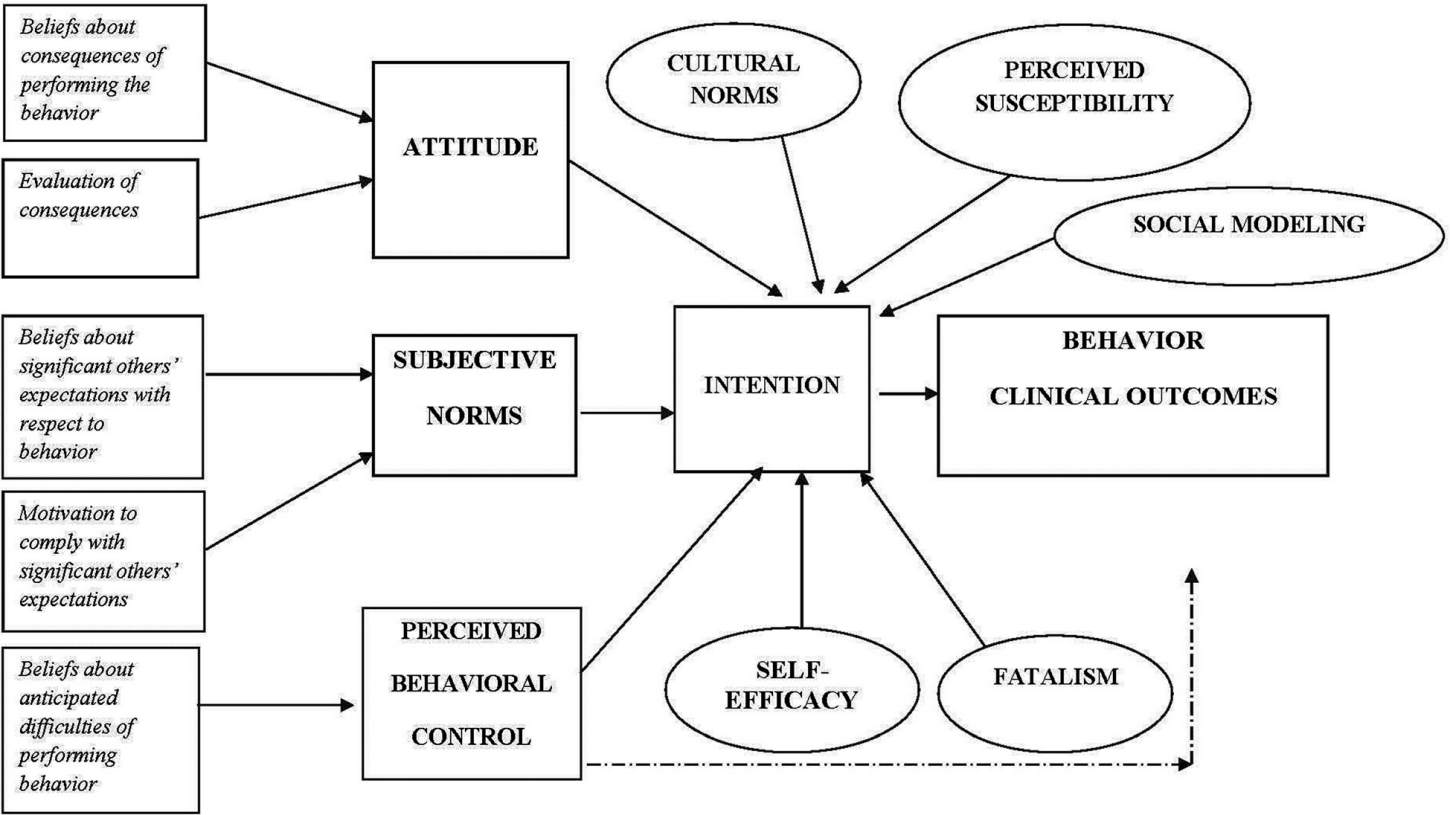
**The proposed expanded model of the Theory of Planned Behavior (TPB).** *Note.* The solid lines refer to a definite direct link between two components, whereas the dotted lines to an indirect link between two components. The squares refer to the TPB constructs and circles refer to additional constructs.

This paper describes a study that is part of a larger project, the aim of which is to develop a theory-based culturally sensitive intervention to promote mammography screening within an AI community in rural Oklahoma. Interventions using a theoretical framework are most effective in increasing breast cancer screening rates [21]. A few studies have used a combination of theoretical models [22,23] by incorporating elements of theories that have been positively associated with mammography behavior. In this study, we also used an integrative conceptual framework that incorporated elements from the Theory of Planned Behavior (TPB) [24,25], Social Cognitive Theory (SCT; self-efficacy, social modeling) [26], the Health Belief Model (perceived susceptibility) [27], and concepts that have been shown consistently to be related to mammography screening such as fatalism [28,29] and cultural norms [9]. The TPB has been used as the primary conceptual model for the development of the assessment survey used in this study [25,30]. The theory posits that intention is the immediate antecedent of behavior and it is assumed to capture the motivation to behave in a particular way. According to the TPB, intention is determined by three factors: attitude toward the behavior, subjective norms (i.e. social norms), and perceived behavioral control (i.e., perceived ease or difficulty of performing a behavior). The theory is based on the assumption that individuals are rational actors and underlying individual reasons determine one’s motivation to perform a behavior, regardless of whether those beliefs are logical, or correct by some objective standard. The strength of the TPB is that it offers a framework for deciphering individuals’ actions by identifying, measuring and combining beliefs that are relevant to individuals and groups, allowing us to understand their own reasons that motivate the behavior of interest. Several studies [31-35] have successfully applied the TPB in mammography screening; however, none of the studies was applied among an AI population. A diagram of the integrated conceptual framework of the TPB is shown in Figure 1.

Although well documented reasons are in the literature regarding as to why AI women do not get regular mammograms limited local research has been conducted to determine why AI women in Oklahoma do not get regular screening mammograms [5]. It is imperative that we conduct research at a local level due to the diversity of AI populations in terms of culture, history and health behaviors across regions and tribes [36]. Moreover, important research questions still need to be answered based on the existing literature, such as what the physician’s role is in mammography screening, and to what degree traditionality or Nativeness influence women’s decision whether to get a mammogram. Furthermore, this is the first study to our knowledge that takes place among AI women who live in a non-reservation setting and attempts to examine whether beliefs identified in the literature of women who live on reservations are relevant to this population. For these reasons, it is vital to examine more fully the influences upon AI women’s decisions to get a screening mammogram within a specific geographical location in Oklahoma.

The primary purpose of this study was to identify the beliefs that AI women who live in a non-reservation setting in rural Oklahoma have regarding regular screening mammography by using the aforementioned integrated model of the TPB. The researchers examined the specific salient motivations related to past mammography experience. More importantly, we wanted to find out which beliefs and health- related practices contributed the most to the explanation of obtaining a recent mammogram.

## Methods

### Study design and participants

Data analyzed for this paper were combined from two independent studies conducted at two points in time using similar methodologies, following the Strengthening The Reporting of Observational Studies in Epidemiology (STROBE) statement, and at the same geographic location [37]. The first study took place from 2005–2006. The purpose of the study was to develop a culturally sensitive survey tailored to the sociocultural environment of the priority population. There were eight major methodological steps: a) review of the published literature to identify beliefs relevant to AI women’s mammography behavior and the constructs of the TPB; b) key informant interviews with breast cancer survivors and clinic representatives (n = 9); c) elicitation interviews with 24 women of the priority population followed by two focus groups; d) development of the first draft of the Women’s Health Survey (WHS); e) review of the WHS by a panel of five experts; f) qualitative review of the survey with two focus group discussions (n = 6); g) pilot-testing of the instrument with a representative sample of the priority population (n = 34) that was reviewed for clarity, readability, comprehensibility, and face validity; and h) establishment of the psychometric properties of the instrument and assessment of the prevalence and relative importance of beliefs identified through the use of a random sample (n = 162). The results of the qualitative piece of the formative research have been published elsewhere [38]. Statistical power was insufficient to perform multivariate statistical analyses due to the limited sample size in the first wave of data collection. Therefore, a second study was conducted from 2011–2012 to collect additional data using the same methodological and random sampling approach (n = 93). A commercial software package was used to estimate the necessary sample size (n = 230) [39]. We assumed that 45% of women in the target population undergo regular screening mammography based on our previous studies. Under this assumption, a total sample size of 230 was required to detect an odds ratio of 2.25 or greater associated with a characteristic that is present among half of the participants, for example, when comparing the odds of screening mammography between groups with Positive Attitude construct scores above and below the median, with 80% power. This calculation assumed a two-sided 0.05 alpha level and assumed that 15% of the variability in the characteristic of interest was explained by the other covariate terms in the regression model to account for potential confounding. Finally, it was assumed that up to 15% of surveys could not be used due to missing data or other errors, resulting in a total target of 271 study participants.

The more recent cohort of patients (n = 93) was older (33% aged 40–49, 42% aged 50–59, and 24% aged 60–69 years) than the earlier cohort (n = 162, 53% aged 40–49, 30% aged 50–59, and 17% aged 60–69 years) (Chi-square test for trend, p = 0.0061) and was more likely to have a positive family history of breast cancer compared to the earlier cohort (42% vs. 20%, Chi-square test, p = 0.0002). No other factors differed significantly between the cohorts. The quantitative data sets from the two waves of data collection were merged and analyzed and are the focus of this manuscript.

### Setting

This study took place at a tribal clinic in Oklahoma among a population of AI women who visited the tribal clinic to obtain health-related services. Potential participants needed to be eligible for screening mammography according to the American Cancer Society guidelines and they were due for the next mammogram within 6 months at the time of entry to the study. The eligibility criteria included women who were asymptomatic of breast cancer and 40–66 years of age. Women were excluded from the study if they worked at the tribal or any other health facility or if they have been diagnosed with breast cancer. The University of Oklahoma Health Sciences Center Institutional Review Board has approved the study.

### Recruitment of participants

The research participants were identified from the computerized list of eligible participants for mammography kept at the clinic. This list included age and prior mammography experience. The participants were assigned a number and were randomly selected using a computerized approach or a table of random numbers. Women were invited by phone or in person to participate in the study by a staff member of the clinic or a member of the research team. The response rate in the first wave of data collection was 84% and, 74% for the second wave of data collection with an average response rate across the two waves combined of 79%. The survey was self-administered at the clinic or in a community setting and its administration took 20–30 minutes long.

### Survey design

The development of the WHS was based on the methodology suggested by the founders of the TPB [24,25] and was completed during the first study from 2005–2006. We defined "regular use of screening mammography" as the self-report of a recent mammogram. Therefore, the dependent variable used for the analysis of this paper (i.e. regular use of mammography screening) was based on the question "when was the last time you had a screening mammogram?" accompanied by a scale with four possible answers "During the past 12 months", "2 years ago", "3-5 years ago" and "more than 5 years ago". For data analysis, a dichotomous outcome variable was defined by combining the first two response categories together to indicate "having a regular mammogram within the last 2 years" and the rest of the answers were grouped together and labeled "not having a regular mammogram".

The independent cognitive constructs included attitude, subjective norms, perceived behavioral control, self-efficacy, breast cancer fatalism, breast cancer susceptibility, social modeling, strength of cultural affiliation, and AI beliefs regarding the AI woman’s role in the current AI society. Each construct was measured by an individual scale and by using the Likert scaling method. Most of the scales were developed by transforming comments derived from the elicitation interviews into item statements; however, three scales were borrowed from other researchers (upon receiving their permission) and adapted to the current study. These scales are the Strength of Cultural Affiliation [40] (19 items), Breast Cancer Fatalism [41] (5 items), and the Breast Cancer Susceptibility scale [42] (6 items). Construct validity was assessed for each component using factor analysis with orthogonal (varimax) rotation. The reliability or internal consistency of each scale was assessed using Cronbach’s alpha values as the reliability estimates. The factor analysis resulted in 15 sub-constructs assessed via 82 belief-statements. Regarding the internal consistency of the sub-constructs, Cronbach’s alphas on the sample ranged from 0.65 to 0.96 (14 out of 15 above 0.70), which indicated that WHS is a reliable instrument. A copy of the survey is available from the first author. The name of each subconstruct and the theory it represents, the corresponding number of items, examples of representative items and the Cronbach’s alpha for each of the scale may be found in Table 1.

**Table 1:** Constructs and representative items

**Name of the construct and its related theory**	**Number of items after factor analysis**	**Representative items after factor analysis**	**Cronbach’s αlpha**
Intention ( TPB)^1^	1	How likely is it that you will obtain a mammogram at a mammography center of your choice within the next 6 months?	N/A
Future Mammography Behavior (TPB)	1	Through medical records and self-reports	N/A
Breast Cancer Susceptibility (HBM)^2^	5	It is extremely likely that I will get breast cancer.	0.86
Positive Attitude ( TPB)	14	Mammography would help me live longer and watch my children and grandchildren grow.	0.96
		Mammography would detect breast cancer early.	
		Mammography would give me peace of mind to find out that I am healthy.	
Negative Attitude ( TPB)	6	Mammography would be wasting my time because mammography cannot detect breast cancer.	0.81
		Mammography would make me uncomfortable because someone else is handling my breasts.	
		Mammography would make me afraid to find out if I have breast cancer.	
Attitude-Mistrust toward mammography (TPB)	2	Getting a mammogram it will be wasting my time because mammography cannot detect small size tumors	0.81
Perceived Behavioral Control (PBC)-Facilitators (TPB)	5	Having someone who sets up the mammography for me would make my getting a regular mammogram easier.	0.75
		Having the mammography facility staff provides me with step-by-step instructions during mammogram would make my getting a regular mammogram easier.	
PBC-Barriers (TPB)	5	It is difficult for me to get my regular screening mammogram because the waiting time in the waiting room at the mammography facility is too long.	0.89
		It is difficult for me to get my regular screening mammogram because the referral process to receive an appointment is too complex.	
Self-Efficacy (scheduling) (SCT)^3^	4	I am confident that I can get a mammogram even if I have to find time to schedule a mammogram.	0.71
Self-Efficacy (procrastination) (SCT)	3	I am confident that I can get a mammogram even though I forget to set up the mammogram appointment	0.79
		I am confident that I can get a mammogram even though I keep putting scheduling the appointment off	
Social Modeling (SCT)	2	If other women know that I get a regular screening mammogram, then they are more apt to go and get a screening mammogram.	0.81
		By getting a mammogram, I feel that I am setting a good example for other women to follow.	
Subjective Norms (family and friends) (TPB)	5	Breast cancer survivors I know think I should get a regular screening mammogram.	0.90
		My children think I should get a regular screening mammogram.	
Subjective Norms (physician) ( TPB)	2	My regular doctor/health practitioner thinks I should get a regular screening mammogram	0.75
		My OB-GYN thinks I should get a regular screening mammogram	
Strength of Cultural Affiliation	16	How much do your home decorations or furniture reflect the influence of your tribe?	0.87
		How often do you follow your tribe’s typical ways in man-woman relationships?	
American Indian beliefs regarding women’s role (leadership role)	3	Native American women are the "pillar" of their families	0.71
		Native American women are the primary caretakers of their families	
		Native American women should be treated with respect and honor	
American Indian beliefs regarding women’s role (traditional role)	2	Native American women should be quiet and reserved	0.65
		Native American women should be separated from others during menstruation	
Breast Cancer Fatalism	5	I think if someone is meant to get breast cancer, they will get it no matter what they do	0.74
		I think getting checked for breast cancer makes people scared that they really have breast cancer	

^1^Theory of Planned Behavior.

^2^Health Belief Model.

^3^Social Cognitive Theory.

Attitude scores were calculated by multiplying the belief strength (that is the perceived strength of association between the belief and its attributes) of each behavioral belief by the outcome evaluation and then summing the products. The perceived outcomes were measured on a 5-point Likert scale, which ranged from "very unlikely" to "very likely". The evaluation of each outcome was measured on a 5-point Likert scale ranging from "Neither good nor bad" to "extremely good" or "extremely bad" depending on the content of the statement [43]. Multiplying the strength of each normative belief by the woman’s motivation to conform to the opinion of significant referents and then summing the results provided a measure for subjective norms. The strength of the normative belief was measured on a 5-point Likert scale, which ranged from "strongly disagree" to "strongly agree". Motivation to comply with the opinion of the referents was measured on a 5-point Likert scale, which varied from "not at all" to "very much". The presence of the perceived behavioral belief/condition was measured on a 5-point Likert scale, which ranged from "strongly disagree" to "strongly agree". The degree of easiness or difficulty to get a screening mammogram if that condition was present was measured on a 5-point Likert scale, which varied from "not at all" to "very much". The self-efficacy scale was measured with items on a 5-point Likert scale which varied from "sure I could not do it" to "sure I could do it". The sum of the product scores across all five items served as the measure of self-efficacy. The Strength of Cultural Affiliation was calculated according to the designer with the scale [38] and the items were measured with 4-point Likert scales with different endpoints depending on the statement including "never" to "always", "very different" to "very similar", "not at all" to "always", "not at all" to "very much", "no one" to "everyone", "makes no difference" to "very much prefer", and "easier" to "more difficult". The rest of the constructs (i.e. breast cancer perceived susceptibility and breast cancer fatalism) were measured with a 5-point Likert scale, which ranged from "strongly disagree" to "strongly agree". The sum of the product scores across all items served as the measure of breast cancer fatalism and breast cancer perceived susceptibility.

### Measures of demographics and clinical characteristics

The WHS also included demographics, clinical characteristics and knowledge of mammography screening. The demographic variables included age, marital status, employment status, income level, educational level, as well as whether the research participants lived in rural areas, and if they had private health insurance. Clinical characteristics included family history of breast cancer, having a primary physician, frequency of visit to the primary physician, frequency of physician breast examination, and frequency of breast-self-examination. All the above variables were treated as categorical variables. Knowledge of mammography screening was measured with four multiple-choice questions with each response coded as correct or incorrect. The number of correct items was totaled to give the knowledge score.

### Data analysis

The two waves of data were combined, resulting in 255 participants. Demographic and clinical history characteristics of the participants were categorized, using standard intervals, and were summarized using counts and percentages. Scale scores were analyzed as continuous measures. Independent sample t-tests were used to compare means of continuous measures between two groups, and Chi-square tests were used to test for an association between categorical measures and to compare proportions. Chi-square tests for trend were used when variable categories were ordered. Logistic regression models were fit to identify demographic, clinical history, and psychological factors associated with the odds of self-reported mammography screening within the last 2 years. Univariate models were fit and factors significant at the 0.1 alpha level, as well as age and education as potential confounding factors, were investigated in a multivariate regression model. The final multivariate model was determined using a backward elimination process in which terms that were not significant at a 2-sided 0.05 alpha level and not indicated to be confounding factors were dropped one-by-one until all factors were significant at a 2-sided 0.05 alpha level or acted as confounders. Confounding was indicated by a 10% or more change in the coefficient estimates of other covariates in the model upon removal of the confounding factor from the model. The confounding factors were included in the final multivariate model. Sub-group effects were not considered. The proportion of missing values was no more than 3% for any of the variables included in the regression model. Therefore, missing data analysis techniques such as imputation methods were not used.

## Results

Demographic and clinical characteristics of the 255 participants are summarized in Table 2. The participants ranged in age from 40 to 66 years with a mean age of 51.34 (SD: 7.64) years. A majority of women (58%) had some post-high school or college education. Slightly over half of the women were unemployed, roughly half were living alone, 30% reported an income of > $45,000, 35% lived in a rural open country area, and 59% had private health insurance. Approximately 91% reported having a primary physician, 66% reported receiving a breast exam by a physician every one to two years, and 89% reported visiting their primary physician at least once per year. Roughly, one-quarter of the women reported a family history of breast cancer. The majority of the research participants (n = 167, 65%) reported that they had a screening mammogram within the last 2 years.

**Table 2:** Demographic and clinical history characteristics of participants (N = 255)

**Variable**	**Levels**	**n**	**Percent**
Age	40-49 years	116	50%
	50-59 years	74	32%
	Over 60 years	43	18%
Have children	Yes	223	87%
Education	Less than high school diploma	37	15%
	Graduated high school or completed GED	68	27%
	Some post high school education	110	44%
	College graduate or more	36	14%
Employment Status	Full-time	100	40%
	Part-time	23	9%
	Unemployed	130	51%
How often have breast exam by physician?	Every year	136	54%
	Every 2 years	30	12%
	Every 3–5 years	42	17%
	Only once/Never	44	17%
Income	0-$15,000	67	27%
	$15,000-$45,000	105	43%
	>$45,000	75	30%
Marital status	Living with someone	131	51%
	Living alone	124	49%
Have a primary physician?	Yes	231	91%
Have a private health insurance?	Yes	151	59%
Rural area	Yes	88	35%
Visit primary physician at least 1/year?	Yes	225	89%
Family history of breast cancer?	Yes	72	28%

The univariate analysis of demographic, clinical history, and psychological (i.e. the integrated TPB constructs) characteristics associated with self-reported history of screening mammography within the last two years is shown in Table 3. Multiple characteristics differed between respondents who did and did not report a mammogram within the last two years. Those participants who reported screening mammography within the last two years had higher levels of income (p = 0.0001), more regular employment (p = 0.0002), and more frequent physician breast exams (p < 0.0001). Respondents who had received a screening mammography within the last two years were more likely to have a primary physician (p = 0.028), private health insurance (p = 0.027) and a family history of breast cancer (p = 0.0005). Mean psychological scale values were higher among women who reported a mammogram within the last 2 years for the perceived behavioral control-facilitator scores (p < 0.0001), subjective norms physician (p < 0.0001), subjective norms-family (p < 0.0001), knowledge (p < 0.0001), positive attitude toward mammograms (p = 0.0004), and social modeling measures (p = 0.0006), but were lower for the perceived behavioral control barriers (p < 0.0001), strength of cultural affiliation (p = 0.0006), negative attitude toward mammograms (p = 0.0011), mistrust toward the effectiveness of mammography screening (p = 0.0091), traditional Native American belief measures (p = 0.0019), and breast cancer fatalism (p < 0.0001).

**Table 3:** Univariate analysis of demographic, clinical history and psychological variables

		**Self-reported screening mammography within last 2 years**		
		**Yes (N = 167)**	**No (N = 86)**	
**Categorical variables**	**Status**	**n (%)**	**n (%)**	**P-value**^**a**^
Age group	40-49 years	71 (43%)	44 (51%)	
	50-59 years	63 (38%)	23 (27%)	0.69
	60-69 years	30 (18%)	19 (22%)	
Education	Less than high school diploma	21 (13%)	15 (18%)	
	Graduated high school or complete GED	45 (27%)	23 (27%)	
	Some post high school education	71 (43%)	38 (45%)	0.14
	College graduate or more	28 (17%)	8 (10%)	
Employment	Full-time	80 (48%)	20 (23%)	
	Part-time	14 (8%)	9 (11%)	0.0002
	Unemployed	73 (44%)	56 (66%)	
How often have breast exam by physician?	Every year	109 (66%)	26 (31%)	<0.0001
	Every 2 years	21 (13%)	9 (11%)	
	Every 3–5 years	24 (14%)	18 (21%)	
	Only once/Never	12 (7%)	31 (37%)	
Income	0-$15,000	33 (20%)	32 (40%)	
	$15,000-$45,000	70 (43%)	35 (43%)	0.0001
	>$45,000	61 (37%)	14 (17%)	
Marital status	Living with someone	92 (55%)	39 (45%)	0.14
	Living alone	75 (45%)	47 (55%)	
Have a primary physician?	Yes	156 (93%)	73 (85%)	0.028
Private health insurance	Yes	107 (64%)	43 (50%)	0.027
Rural areas	Yes	60 (36%)	27 (32%)	0.49
Visit primary physician at least 1/year?	Yes	150 (90%)	73 (85%)	0.20
Family history	Yes	58 (35%)	12 (14%)	0.0005
**Continuous variables**		**Yes**	**No**	
		**Mean (SD)**	**Mean (SD)**	**P-value**^**b**^
Perceived susceptibility toward breast cancer		2.51 (0.92)	2.46 (0.95)	0.66
Perceived behavior control-facilitator		17.31 (5.72)	13.99 (6.33)	<.0001
Perceived behave control-barrier		3.87 (3.55)	7.75 (5.33)	<.0001
Self-efficacy- scheduling		4.40 (0.85)	4.21 (0.90)	0.10
Self-efficacy -procrastination		4.01 (1.03)	3.85 (1.01)	0.26
Subjective norms-physician recommendation		22.47 (4.42)	17.34 (7.08)	<.0001
Subjective norms-family influence		20.74 (4.99)	17.45 (6.42)	<.0001
Strength of cultural affiliation		1.12 (0.74)	1.48 (0.88)	0.0006
Knowledge of mammography screening guidelines		3.04 (1.00)	2.31 (1.26)	<.0001
Positive attitude toward mammography		21.77 (4.34)	19.52 (5.43)	0.0004
Negative attitude toward mammography		5.27 (3.34)	7.09 (5.35)	0.0011
Negative attitude-mistrust		6.17 (4.27)	7.89 (5.95)	0.0091
Social modeling		4.22 (0.84)	3.78 (1.15)	0.0006
Native American beliefs (leadership role)		3.92 (0.79)	4.09 (0.88)	0.13
Native American beliefs (traditional role)		1.84 (1.02)	2.30 (1.28)	0.0019
Breast cancer fatalism		11.42 (4.04)	14.4 (5.18)	<.0001

^a^Proportions compared between groups using a Chi-square test and a Chi-square test for trend when response options included more than two ordered categories. ^b^Means compared between groups using an independent sample t-test.

Table 4 includes a summary of the final multivariate logistic regression analysis of the odds of self-reported screening mammography in the last two years after adjustment for age and education, which acted as confounding factors. The odds of reporting a past mammogram within last 2 years were higher for the women who were more likely to be influenced by their physicians (OR = 1.15, 95% CI: 1.06-1.24), had more knowledge about mammography screening guidelines (OR = 1.52, 95% CI: 1.00-2.31), had a family history of breast cancer (OR = 9.97, 95% CI: 3.05-32.62), and had more frequent professional breast exams (every 3–5 years with OR = 1.68, every 2 years with OR = 3.67, every year with OR = 5.57). The odds were lower for the women who were more culturally affiliated (OR = 0.42, 95% CI: 0.24-0.74), had higher fatalism scores (OR = 0.90, 95% CI: 0.82 to 0.90), and had higher perceived behavioral control barrier scores (OR = 0.86, 95% CI: 0.78 to 0.94).

**Table 4:** **Multivariate logistic regression analysis**^**a **^**of the odds of self-reported screening mammography within the last two years**

**Parameter**	**Level**	**Estimate**	**Standard error**	**Odds ratio (OR)**	**Lower 95% CI for OR**	**Upper 95% CI for OR**	**P-value**
Intercept		-0.41	1.38	0.66	0.04	9.98	0.77
Subjective norm-physician		0.14	0.04	1.15	1.06	1.24	0.0007
Cultural affiliation		-0.86	0.29	0.42	0.24	0.74	0.0026
Fatalism sum		-0.10	0.05	0.90	0.82	0.99	0.035
Knowledge		0.42	0.21	1.52	1.00	2.31	0.047
Perceived behavior control-barrier		-0.16	0.05	0.86	0.78	0.94	0.0015
Family history (Ref = No)	Yes	2.30	0.60	9.97	3.05	32.62	0.0001
How often have breast exam by physician (Ref = only once/never)	Every 3–5 years	0.52	0.70	1.68	0.42	6.67	0.46
	Every 2 years	1.30	0.76	3.67	0.82	16.41	0.089
	Every year	1.72	0.58	5.57	1.79	17.37	0.003

^a^Multivariate model adjusted for age and education.

## Discussion

The purpose of the study was to identify factors predictive of regular mammography screening among AI women who lived in a non-reservation setting in rural Oklahoma. The results of this study, along with the results of follow-up qualitative research, are being used to plan an intervention to promote mammography screening in this particular region.

One important question that this study attempted to answer was the role that the physician plays in AI women’s decision to get a screening mammogram. To the best of our knowledge, this is the first study among AI women that provided clear evidence that physician recommendation is an important motivating factor of screening mammography. Furthermore, research participants who had a physician breast examination every year were roughly *six times* more likely to get a recent mammogram than those who never had a physician breast examination or who had only once in their lifetime. This finding underscores the importance of the physician as a gatekeeper in the promotion of screening mammography as supported by the literature [12-15]. This finding also indicates the importance of having in place clinic-based policies and procedures, such as a checklist of possible topics to discuss with the patient about mammography screening during her clinic visit, which will facilitate and support a physician recommendation.

The role that the clinic plays also is emphasized though the fact that women who perceived fewer environmental behavioral control barriers, related to the referral process, scheduling a mammogram, and waiting for an appointment, were more likely to get a mammogram. This underscores the importance of the clinical setting and that it must be conducive to mammography screening. Most of the research conducted in this area among AI women has focused on the lack of access to mammography screening or lack of culturally sensitive care [6,19]. In our study we were able to identify specific barriers related to scheduling and referral processes of mammography screening as well as barriers related to the facilities where the mammography is taking place. Overall, the women who utilize the services of the clinic are satisfied with the quality of services provided, which included the scheduling and referral services for mammography screening. Nevertheless, opportunities likely exist to build upon and enhance a clinic team-based approach to promote breast cancer screening among patients. The Centers for Disease Control and Prevention (CDC) in collaboration with the American Cancer Society provides an evidence-based framework for promoting cancer screening in clinical settings [44]. The guide, which may be used as a template, suggests to increase cancer screening, physicians, nurse practitioners, physician assistants and their office managers collaboratively focus on developing a systematic approach toward physician recommendation, supportive office policies (e.g. an algorithm of current mammography screening recommendation guidelines), effective reminder systems, and skillful communications such as the use of decision aids.

Regarding the barriers related to the mammography facilities, one has to keep in mind, that the study took place at a tribal clinic where mammograms are scheduled in other facilities through the contract health department of the clinic. Therefore, working with the various facilities in the region where women get their mammograms is beyond the scope of this study; however, it is useful for the clinic officials to be aware of any factors (e.g. long waiting hours) associated with the mammography facility because those factors can affect a woman’s decision to get a mammogram.

Another interesting perceived barrier found was that women who kept putting off scheduling a mammogram were less likely to get a mammogram. Procrastination is an indicator of an underlying fear, which also has been captured through prior qualitative research conducted by our team [38]. A possible consequence of the presence of underlying fear is that women may not show up at the mammography facility even if they have scheduled their mammograms. The problem of "no-shows" is an important issue for clinics nationwide and account for 13% to 23% of scheduled appointments [45,46]. Based on the first preliminary study conducted back in 2005–2006 some of the reasons women cited for not showing up for their mammogram appointments were expected, such as last minute conflicts. However, other unexpected reasons arose such as not wanting to get a mammogram in the first place and perhaps being afraid of voicing it out to the medical staff.

Similar to the fear concept is fatalism. AI women who expressed more fatalistic attitudes toward mammography and breast cancer were less likely to get regular mammograms. Cancer fatalism is the belief that an individual’s health is beyond their control and that survival is based on luck, fate, and destiny [47]. A woman’s perception of cancer as a death sentence may hinder her from seeking early detection, early diagnosis and treatment. Limited studies examining breast cancer fatalism exist especially among AI women [5,48]. Fatalistic beliefs have been associated with the avoidance of cancer-related information. In one study, researchers found that fatalistic beliefs were correlated with being less positive about early cancer detection and more fearful about seeking help for a suspicious symptom [49]. With regards to breast cancer screening, studies have indicated that after controlling for education and economic status, fatalism was prevalent among poorer and less educated populations [50]. To understand breast cancer fatalism and its impact on early diagnosis screening among AI women, it is vital to address the interrelationship between fatalism and the web of poverty. Within the AI community, historical issues of political and social conflicts are often the root of social inequalities. The disparities existing within AI community members have affected the health care sector especially in the fight against chronic diseases such as cancer. Inadequate access to quality health care, medical mistrust of Western medicine, and a lack of understanding about cancer, screening, and treatment are some of the leading factors that affect cancer health experiences and views. Fatalism, along with fear, is a perception that is formed for a long time through one’s sociocultural context and it is too difficult to uproot within the limitations of a 3-year project. On the other hand, fatalism in this study was marginally negatively associated with age (p = 0.08), but significantly negatively associated with knowledge about mammography screening (p = 0.008). Therefore, one possible way to counteract fatalism is by providing knowledge about mammography screening, specifically information on survival rates, and by having AI women who are breast cancer survivors share their experiences with other women, especially younger women whose beliefs and feelings are more amenable to change. By doing that, we will "plant the seeds" for future efforts taken by the broader AI community that will enable AI women to overcome their fatalistic attitudes about breast cancer and become less fearful in terms of getting regular mammograms.

The importance of knowledge of mammography screening is further highlighted in the results of this study and supported by the existing literature [5]. Knowledge is a necessity for any behavioral change but not sufficient to promote behavioral modification especially when it takes place in a community setting [51]. According to the social ecological model [52-54], to develop sustainable and effective interventions, all levels of intervention must be addressed including the intrapersonal (e.g. knowledge), interpersonal (e.g. social modeling), community (e.g. educational events), policy (e.g. use of a flowchart of the current mammography screening recommendation guidelines) and societal (e.g. change in the cultural norms).

Another unique finding is the more traditional a woman was in terms of her Nativeness, the less likely it was that she would get a recent mammogram. This finding sheds some light onto an existing debate in the literature regarding the role Native identity plays in getting a regular mammogram. Interestingly enough, after conducting some additional data analysis, no statistically significant association was seen between the strength of cultural affiliation neither with age nor with fatalism. However, based on additional data analysis, we found that cultural affiliation is associated with employment status and income levels. Unemployed and lower income women were more likely to be more traditional. Therefore, unemployment and income may confound the association between cultural affiliation and recent mammogram behaviors. Nevertheless, the results of the study are clear; the more traditional AI women were, the less likely it was that they had a recent mammogram. One possible explanation for this finding is that women with higher cultural affiliation may be more likely to rely on traditional medicine and not value preventative medicine as a way of maintaining health. Other possible reasons, as they were expressed in the literature, are that traditional women may not get mammograms due to feelings of invasion, modesty, and mistrust or fear of using the Western health care system [5,8]. Therefore, to reach AI women who are more traditional in terms of their native identity, it is important to try to change their overall perceptions about the health care system, regarding not only care, but also prevention. This task could be difficult to achieve because of the long lasting impact of the historical trauma these women experienced.

This study has several limitations. The health behavior information, including frequency of mammography, was based on self-report, which may not be accurate. There is a potential for sampling bias, due to non-response bias, given that not all women who were invited to participate actually agreed to complete a survey; however, with a response rate of 79%, non-response bias is not a major concern. Results from the sample of AI women who lived in a non-reservation setting in rural Oklahoma are reflective of the beliefs, knowledge and health practices of the targeted population and may not be generalizable to all AI women.

## Conclusion

Breast cancer is an important public health issue among AI women. The results of this study shed some light on approaches that can be taken to intensify efforts to promote mammography screening, the single most effective modality of early breast cancer detection, among AI women who live in a non-reservation rural setting. Emphasis could be given on promoting physician recommendation, physician breast examination and eliminating environmental barriers. Fatalistic attitudes toward breast cancer do exist among AI women. As such, long-term interventions within the AI communities may be appropriate to reduce or eliminate breast cancer-related fatalism and fear and to increase knowledge about breast cancer screening guidelines.

## Abbreviations

AI: American Indian; CDC: Centers of disease control and prevention; CI: Confidence intervals; OR: Odds ratio; SCT: Social cognitive theory; SES: Socio-economic status; STROBE: Strengthening the reporting of observational studies in epidemiology; TPB: Theory of planned behavior; WHS: Women’s health survey.

## Competing interests

The authors declare that they do not have any competing interests.

## Authors’ contributions

ET contributed to the conception and design of the study and the acquisition, and interpretation of the data, drafted the manuscript, and gave final approval of the manuscript version submitted for publication. JS contributed to the design of the study, analysis, interpretation of the results, drafted part of the manuscript, reviewed critically the manuscript, and gave final approval of the manuscript. LJ contributed to the analysis and interpretation of the results. She also drafted part of the manuscript, reviewed the manuscript and provided feedback, and approved the final version of the manuscript. YK contributed to the design of the study, acquisition of data, data management, reviewed critically the content of the manuscript and gave her final approval of the manuscript. KE contributed to the design of the manuscript, interpretation of the results, contributed significantly to the intellectual content of the manuscript and gave her final approval of the manuscript.

## Pre-publication history

The pre-publication history for this paper can be accessed here:

http://www.biomedcentral.com/1472-6874/14/101/prepub
